# Regulatory and Industry Perspective on the Model Master File Framework for Locally Acting Drug Products

**DOI:** 10.1007/s11095-025-03823-5

**Published:** 2025-02-26

**Authors:** Ross L. Walenga, Khondoker Alam, James F. Clarke, Jan De Backer, Markus Fridén, Abdullah Hamadeh, Jay Mowli, Sujatha Sonti, Jessica Spires, Ming-Liang Tan, Flora T. Musuamba, Eleftheria Tsakalozou

**Affiliations:** 1 Division of Quantitative Methods and Modeling, Office of Research and Standards, Office of Generic Drugs, Center for Drug Evaluation and Research, U.S. Food and Drug Administration, White Oak Building 75, Room 4700, 10903 New Hampshire Avenue, Silver Spring, MD 20993 USA; 2 Certara Predictive Technologies Division, Certara UK, Sheffield, UK; 3 FLUIDDA INC., New York, NY USA; 4 Inhalation Product Development, Pharmaceutical Technology & Development, AstraZeneca, Gothenburg, Sweden; 5 University of Waterloo, Waterloo, Canada; 6 Capstone Development Services Co, LLC, Rosemont, IL USA; 7 GSK, Collegeville, PA USA; 8 Simulations Plus, Inc., Lancaster, CA USA; 9 Clinical Pharmacology and Toxicology Research Unit, University of Namur, Namur, Belgium; 10 Federal Agency for Medicines and Health Products, Brussels, Belgium

**Keywords:** computational fluid dynamics (CFD), locally acting drug products, model master file, physiologically based pharmacokinetic (PBPK)

## Abstract

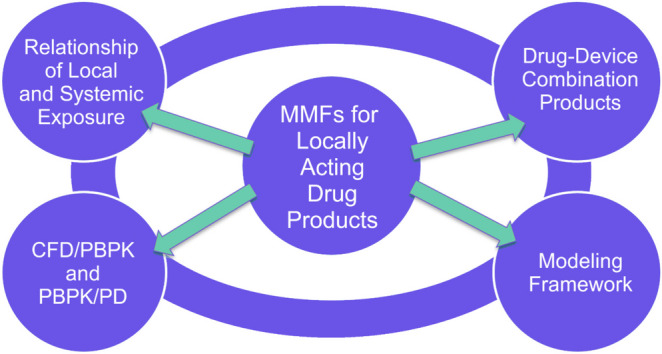

## Introduction

The Model Master File (MMF) concept has been discussed in several public forums including two public workshops co-hosted by the U.S. Food and Drug Administration (FDA) and the Center for Research on Complex Generics (CRCG) [1, 2]. Experts from the FDA and other regulatory agencies, new and generic drug developers, academic institutions, contract research organizations (CROs), software vendors, consultants, and others involved in drug product development have engaged in conversations on the MMF concept, its utility and potential applications. The diverse community participating in these interactions debated challenges with the implementation of the MMF in the current drug development ecosystem. To promote progress on the MMF initiative, a recent publication by Fang *et al*. [3] offered a comprehensive account on the MMF concept including a definition and modeling and simulation (M&S) case studies that could be serve as MMF applications. In highlighting the regulatory context of the MMF initiative, M&S applications that supported regulatory (full or tentative) approvals were presented. These case studies involved validation of a modeling framework for drug products applied on the skin, model-based data imputation methodology developed to address issues of data sparsity often seen with certain drug products, and an adequately validated oral absorption model that mitigated the risk of BE failure due to variation in certain quality aspects identified during the regulatory assessment for an oral dosage form. Additional considerations were presented on model shareability and reusability in the public domain or on how to safeguard proprietary information and intellectual property.

To further explore the concept of an MMF via conversation among members of FDA, the pharmaceutical industry, and academia, a workshop with the title “Considerations and Potential Regulatory Applications for a Model Master File” was organized by CRCG and FDA and was hosted in Rockville, MD, USA from May 2-3, 2024 [4]. The overall aim of this workshop was to increase efficiency for development of M&S approaches to support drug product development via the MMF concept. There were several sessions over the two-day workshop covering a variety of topics including the MMF framework, MMF applications for oral drug products, long-acting injectable drug products, locally acting drug products, and potential pathways for regulatory acceptability of MMFs in new and generic drug spaces. The subject of this publication is the use of MMFs for M&S of locally acting drug products, which was covered on Day 2 in the session with the title “MMF Applications for Locally Acting Drug Products.”

Locally acting drug products include those that are delivered upstream of systemic circulation to various tissues in the body via a variety of routes of administration, including, but not necessarily limited to, buccal, dermal, gastrointestinal, vaginal, intrauterine, nasal, orally inhaled, ophthalmic, and otic routes of administration. To support development and approval of locally acting drug products, mechanistic models may be used to quantify drug delivery to the site of action. The advantage of model predictions for drug concentration at the site of action is that *in vivo* methods are incapable of quantifying these values on a routine basis, while *in vitro* physicochemical characterization and *ex vivo* methods only provide indirect assurance of bioequivalent drug delivery to the site of action. Even for cases where *in vivo* relevant local concentration data are available (such as the lung or the aqueous humor of the eye), there are challenges with obtaining robust measurements and appropriately interpreting the drug amounts in the samples in terms of pharmacologically active concentration. Thus, mechanistic models may provide a means for bridging the gap between drug concentration at the site of action and available *in vivo*, *ex vivo*, and *in vitro* data that may not provide direct observations of local tissue concentration. However, in some cases, development of such mechanistic models may be resource intensive. To alleviate the burden of model development for drug product development and approval, the concept of the MMF was discussed in relation to mechanistic models of locally acting drug products at the CRCG workshop mentioned above, via six presentations, a panel discussion, and small group discussion, which are summarized in the text below, and are also available in the form of recordings and slides [4, 5].

## Presentations

### Regulatory Perspective on MMF Applications for OIDPs, Ophthalmic Drug Products, and Drug Products Applied on the Skin

**Ross Walenga, PhD**

**FDA**

Dr. Ross Walenga (FDA) introduced a series of proposed model applications that may represent useful MMF examples for generic locally acting drug products, including orally inhaled drug products (OIDPs), ophthalmic drug products, and drug products applied on skin [6]. The purposes for modeling of generic OIDPs were identified as acceleration of product development and justification of biorelevant bioequivalence (BE) limits for relevant *in vitro* studies, as described in the recently published new draft product-specific guidance for formoterol fumarate; glycopyrrolate inhalation metered aerosol [7]. The first of two examples was expanded upon, which was based on computational fluid dynamics (CFD) regional deposition modeling for metered dose inhalers (MDIs), where the specified purpose was to identify biorelevant BE limits for realistic aerodynamic particle size distribution (APSD) testing. In this case, the MMF may include information related to model validation as well as selection of physical models, model inputs such as *in vitro* realistic APSD and plume geometry data, mesh density and time step duration, and the human airway geometry. Dr. Walenga proposed that such an MMF may only be applicable for other MDIs, besides the MDI used to develop the MMF, when no significant formulation differences are present. For instance, it may not be appropriate to apply an MMF developed for a solution-based MDI to a suspension-based MDI, or to apply an MMF developed for a an OIDP with a single active ingredient to an OIDP with multiple active ingredients. For the second example, an intravenous (IV) physiologically based pharmacokinetic (PBPK) model for a drug substance in an OIDP was identified as a potential MMF application. The clearance and distribution parameters determined based on the IV PBPK model may then be included in the putative MMF, which may then be applied to PBPK modeling for any other drug product of the same drug substance, independent of the route of administration or dosage form.

Purposes for PBPK modeling of generic ophthalmic drug products were described as correlating *in vitro* metrics to *in vivo* exposure, conducting virtual BE simulations in the target eye tissues, and informing product development via interspecies model extrapolation supported by preclinical data. An MMF application was proposed for validated drug diffusion and partitioning components of ophthalmic PBPK models, such that these components may be re-used across different dosage forms such as solutions, suspensions, and emulsions. To illustrate this application, PBPK predictions following administration of dexamethasone ophthalmic suspension in rabbits from Le Merdy *et al*. [8] were discussed. These predictions were based in part on diffusion and partitioning drug parameters [8], which were then successfully applied to a PBPK model for dexamethasone ophthalmic ointment in a subsequent study [9]. A second example of an MMF application for ophthalmic drug products was also introduced, which outlined the potential for using a validated ocular organ model across multiple products. Validation of the eye model would be used to ensure that selected anatomical and physiological parameters may be used for new cases without the need for justification, where these values may include for tear film and tissue thickness, surface area, tissue volume, tear fluid turnover rate, and melanin level.

**Fig. 1:**
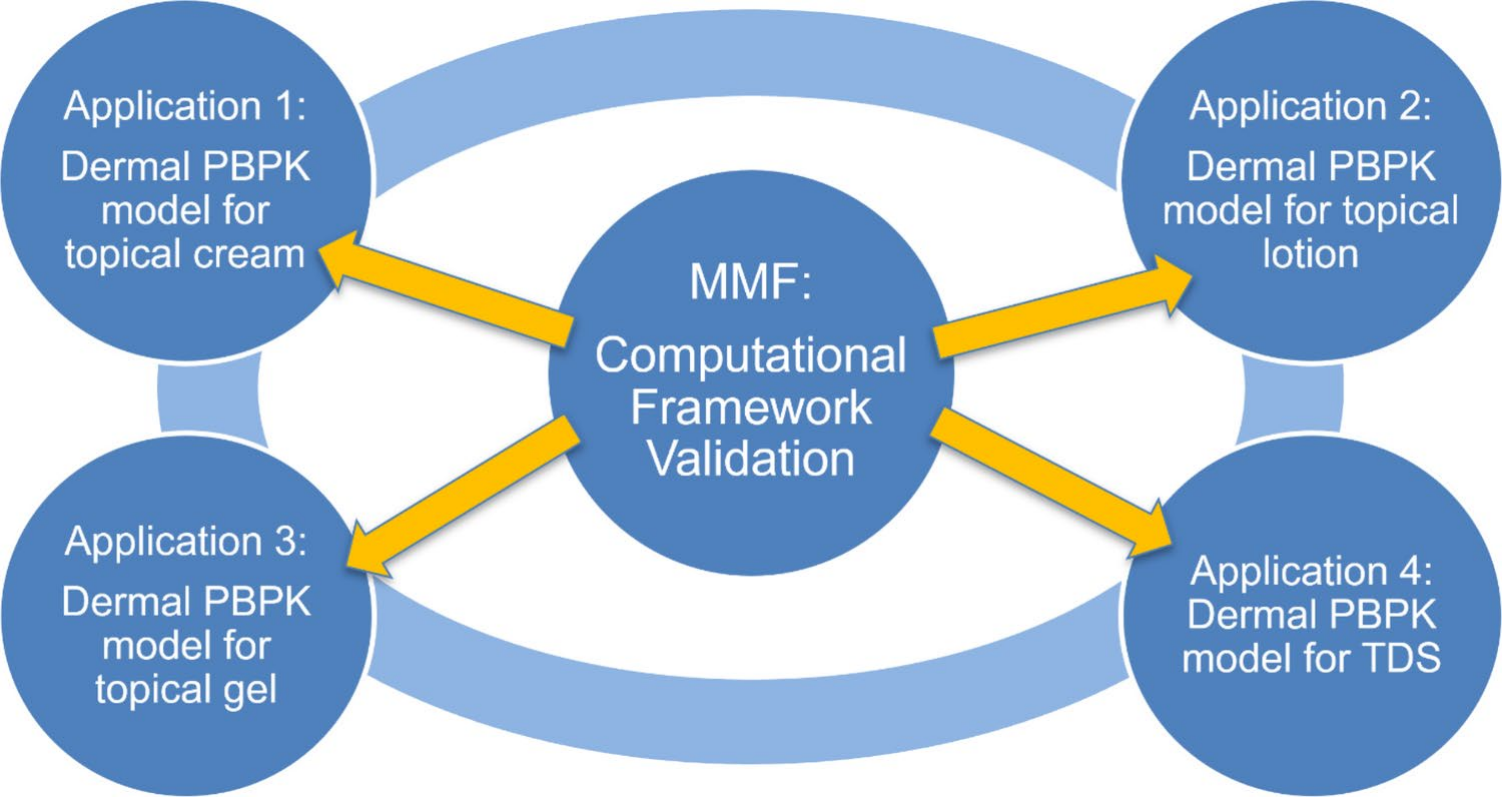
Flowchart of how platform validation of a dermal PBPK model, such as the platform validation described in Tsakalozou *et al*. [10], may be used to support an MMF that may then be applied for various dosage forms, including topical creams, topical lotions, topical gels, and transdermal systems (TDSs).

Regarding generic drug products applied on the skin, Dr. Walenga introduced potential purposes for using PBPK modeling, which included identification of a “safe space” for *in vitro* quality metrics for the reference standard (RS) where variation of these metrics within that “safe space” would confine *in vivo* performance to a pre-specified set of acceptable limits. Other purposes included prediction of skin permeation as represented with *in vitro* permeation test (IVPT) results, as well as assessment of BE in systemic circulation and/or the skin between RS and test products, for circumstances when *in vivo* testing may be difficult. One proposed MMF application was for modeling framework validation of a PBPK model intended for prediction of absorption through the skin, as illustrated by the results of Tsakalozou *et al*. [10]. For that study, a PBPK model was developed for diclofenac sodium topical gel that was both validated on its own and supported by validation of the modeling framework it was built in, that included over ten active ingredients, seven dosage forms, and seven biological matrices for validation [10]. Such a validated modeling framework could then be used across multiple dosage forms, as illustrated by Fig. 1. The results from Duong *et al*. [11] were then used to introduce another proposed MMF application, which was described as development of an *in vitro* *in vivo* extrapolation (IVIVE) methodology that utilized IVPT data to construct an *in vivo* dermal PBPK model. For this example, the development of a ketoconazole topical cream PBPK model used to determine a “safe space” for relevant quality attributes was detailed, which included development of an oral PBPK model to describe systemic disposition and a skin absorption model to describe skin permeation for ketoconazole, as well as the use of IVPT data to determine influential model parameters [11].

### EMA Experience with Qualification of Modelling and Simulation Methods

**Flora Musuamba, PhD**

**University of Namur; Belgian FAMHP**

To provide global context on the proposed use of MMFs, Dr. Flora Musuamba (University of Namur; Belgian Federal Agency for Medicines and Health Products [FAMHP]) delivered a presentation that focused on the Qualification of Novel Methodologies program offered by European Medicines Agency (EMA) [12]. Several avenues are available for the public to discuss novel methodologies with EMA, including the Innovation Task Force (ITF), Scientific Advice, Qualification of Novel Technologies (including qualification advice and qualification opinion procedures), and Marketing Authorisation Application. The Qualification of Novel Methodologies program is intended to provide early communication with interested parties on the type and quantity of evidence required to support the use of a novel methodology for a given context of use (COU), which are referred to as qualification procedures [13]. These interested parties may include consortia, CROs, and pharmaceutical companies, among others. Two types of feedback are offered with the Qualification of Novel Methodologies program, including the Committee for Medicinal Products for Human Use (CHMP) Qualification Opinion and CHMP Qualification Advice pathways. The CHMP Qualification Opinion procedure provides feedback on a given COU based on available data. Most commonly the outcomes of positive qualification opinion are applicable to a wide range of drug development programs for the concerned drug development question and context of use. When positive, the CHMP qualification opinion is then made publicly available on the EMA website [14, 15], while the CHMP Qualification Advice pathway provides confidential feedback on future methods and how these may be qualified for the intended COU. Once the CHMP Qualification Opinion has been published on the EMA website, it may be referred to in future applications for the same COU [14, 15]. Altogether, qualification procedures are encouraged by EMA to support the use of M&S tools, especially for high regulatory impact applications (i.e., when the model is used to replace the established source of evidence such as a clinical study). Good candidates for EMA qualification procedure include modeling platforms that are intended to be used across a variety of drug products, and complicated M&S tools that are developed using retrospective data across several drug development programs.

Over the past five years, the total number of requests for Modelling and Simulation-related Qualification Opinion and Qualification Advice has risen from three in 2019 to four in 2022, and then to seven in 2023, where the requests originated from subject matter experts, consortia, and the pharmaceutical industry. The scope of these requests includes pre-clinical development applications such as supporting waiver of components of non-clinical studies, clinical development applications such as dose finding, population enrichment, and surrogate endpoints, as well as drug utilization to optimize the target population and guide treatment regimens. In most cases, a letter of support or final advice letter was issued to the applicant for instances when further model development was needed, but in some cases a positive opinion was concluded when the model was deemed acceptable for the proposed COU. Many of the problems identified from submitted requests include inadequate COU definition, issues with data quality, missing information and poor reporting, and inappropriate methodological implementation.

Two examples of requests for Qualification Opinion and Qualification Advice were discussed, including a model-based tool for dose selection of drugs used to treat osteoporosis and the use of islet autoantibodies (AAs) as enrichment biomarkers for Type 1 diabetes (T1D) prevention clinical trials. For the case with the dose selection tool for drugs used to treat osteoporosis, EMA identified uncertainties in the methodology and communicated an unfavorable view on the applicant’s plan to use the model without model validation based on clinical data. Rather, it appeared that the applicant’s motivation for submitting the request was to illustrate the EMA regulatory process of qualification of mechanistic models to support drug development and related rechallenges. Given this and given the major limitations identified, the CHMP determined that an in-depth technical discussion would was not warranted. In contrast, for the case proposing the use of islet AAs as enrichment biomarkers for T1D prevention clinical trials, the applicant worked with EMA to refine their COU, method, and data sources such that EMA ultimately provided a positive opinion that is now available on the EMA website [15].

### Advancing Orally Inhaled Products Through Digital Twins and In-Silico Trials: Strategies for Model Master File Creation

**Jan De Backer, MSc, PhD, MBA**

**FLUIDDA INC.**

In this presentation, Dr. Jan De Backer (FLUIDDA INC.) emphasized the role of MMFs for the advancement of OIDPs via digital twins and in silico trials [16]. The COU that was considered for this presentation was to use in silico models alongside *in vivo* pharmacokinetics (PK) and *in vitro* studies to obtain bio-waivers for comparative clinical endpoint BE studies to support the approval of generic OIDPs. Key drivers for regional exposure must reflect real clinical settings, with a particular focus on central versus peripheral deposition in the relevant patient populations.

**Fig. 2:**
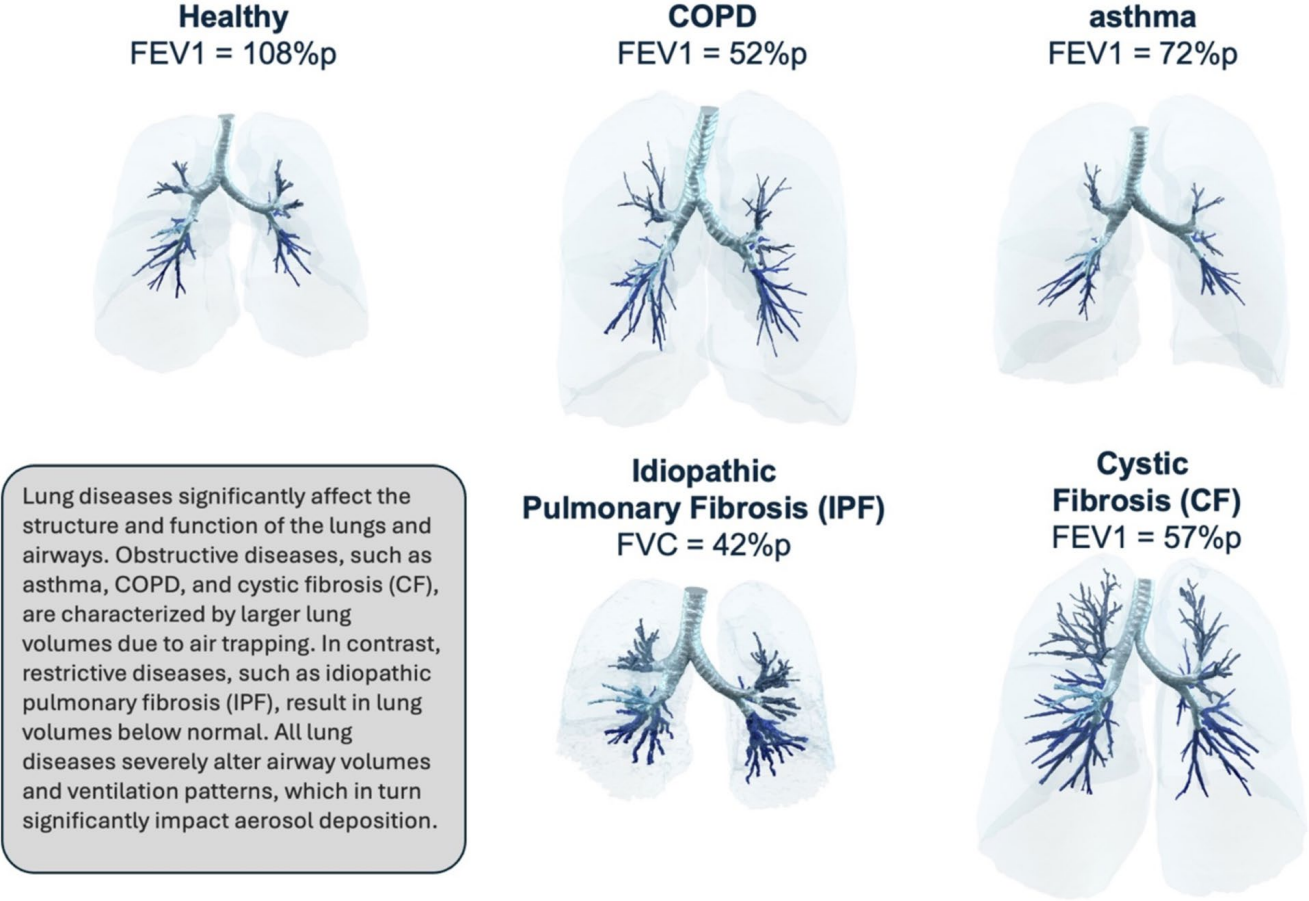
Lung diseases severely alter lung structure and function, significantly impacting aerosol deposition. For in-silico methods to be a reliable digital equivalent of a clinical trial, they must include relevant patient-specific features.

A proposed approach for supporting the COU was then detailed by Dr. De Backer. For the described approach, CFD plays a crucial role by solving the equations that describe flow behavior, where its accuracy depends in part on input parameters that are intended to represent actual clinical scenarios. To support accurate model input parameters, the use of Functional Respiratory Imaging (FRI) was proposed, which involves the use of computed tomography (CT) scans in combination with CFD to produce quantitative outcome parameters without radiolabeling pharmaceutical aerosols. Such use of FRI offers detailed insights into drug deposition, lung volumes, airway volumes, and other critical metrics. Studies have shown the effectiveness of FRI in assessing total lung deposition compared to traditional methods like scintigraphy [17–31]. The necessity of including diseased lungs and airways in models was underscored (Fig. 2), to ensure accurate representation of conditions like chronic obstructive pulmonary disease (COPD), asthma, cystic fibrosis, and idiopathic pulmonary fibrosis [32]. The modeling approach may be validated using existing evidence for total lung dose and lobar dose [30, 31], supported by prospective cross-over studies. These validation studies involve different formulations, healthy volunteers, diseased patients, and methods like gamma scintigraphy and FRI deposition based on high-resolution CT scans. Once the modeling approach is validated, Monte Carlo simulations may be employed to help with understanding the impact of varying input parameters (e.g., fine particle fraction, airway volume, inhalation flow) on lung and local deposition.

Dr. De Backer concluded that the presented OIDP modeling approach ensures accurate modeling of inhaled drug deposition, which may be applied for the development of effective respiratory therapies. This modeling approach may be the subject of an MMF, which may be a useful tool for streamlining the approval process of complex generics such as OIDPs, especially with advances in digital twins, in silico trials, and artificial intelligence. A well-prepared MMF should capture all clinically relevant parameters and demonstrate its effectiveness within its COU.

### Physiologically Based Biopharmaceutical Modelling in Virtual Comparative Clinical Endpoint Studies of Orally Inhaled Drugs

**Markus Fridén, PhD**

**AstraZeneca**

Dr. Markus Fridén (AstraZeneca) discussed the potential use of physiologically based biopharmaceutics modeling (PBBM) to provide robust and precise assessment of drug product comparability at the level of comparative clinical endpoints, aiming to provide an alternative to conducting large and complex clinical studies to investigate the impact of drug product differences [33]. The concepts of linking PBBM (or PBPK) to local lung pharmacodynamic (PD) biomarkers of drug response has been described previously [34–36] and has become common practice in drug discovery and development to interpret *in vivo* study results and inform decision-making on study designs and program progression. The principle is that when a pharmacokinetic/pharmacodynamic (PK/PD) relationship exists between the drug exposure at the relevant lung target site and the local PD biomarker, the model can be used to evaluate the impact of formulation and/or device changes on the biomarker or clinical endpoint. Provided that there are adequate model verification and validation for the COU, it was proposed that virtual comparative clinical endpoints would offer advantages over clinical studies in terms of speed of discovery, statistical powering, and, most importantly, avoiding additional burden due to a large number of patients.

**Fig. 3:**
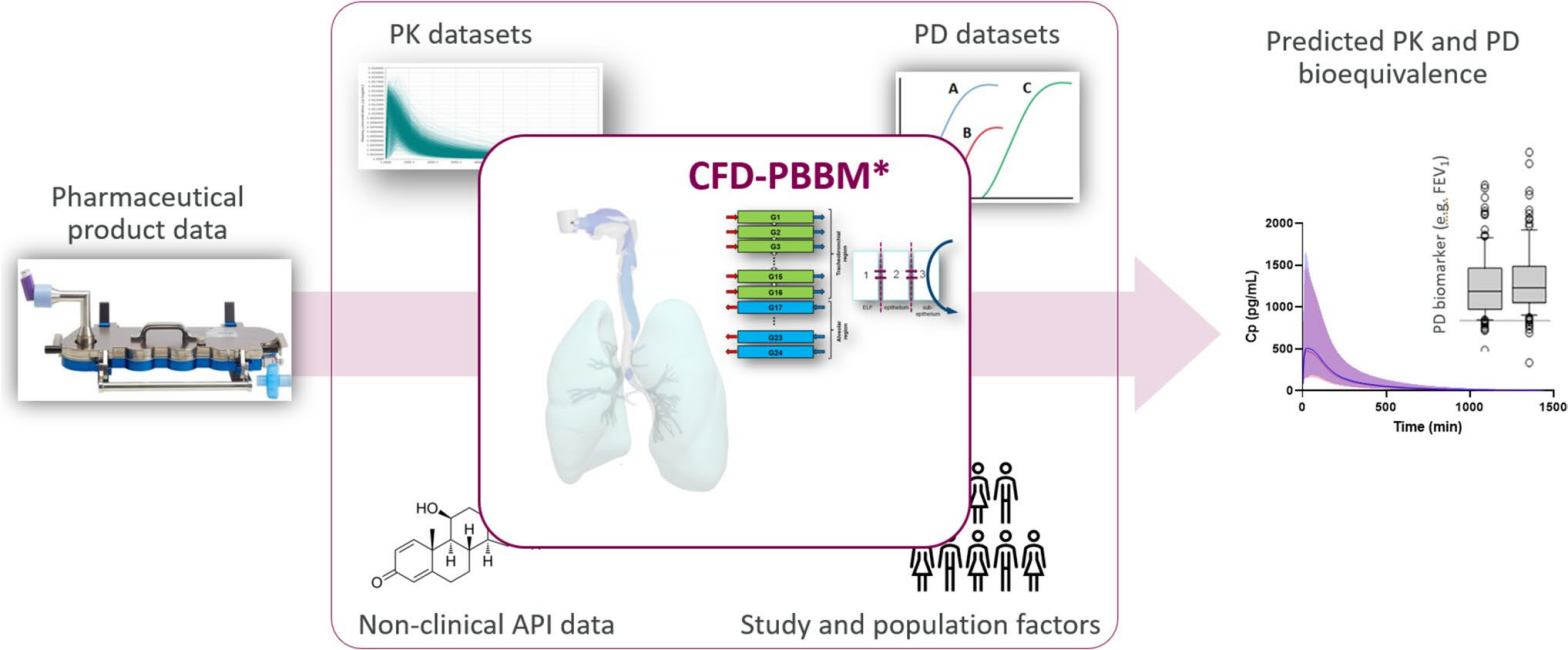
CFD-PBBM model links changes in drug product to clinical PK and PD bioequivalence.

To address the potential role for an MMF in this context, a case study was presented relating to the transition to low global warming potential (LGWP) propellants for MDIs. In brief, the presented model workflow was based on CFD simulations linked to a lung and a whole body PBBM model parameterized with a multitude of clinical and non-clinical datasets along with some propellant-specific *in vitro* datasets. The model accepts batch-release product data as inputs to make predictions of PK BE in plasma and regionally in the lung, where the latter provides the proposed link to the PD biomarker (Fig. 3). Taking AstraZeneca´s portfolio as an example, there can be multiple product brands in one formulation platform (here, Aerosphere® technology), which gives an opportunity to develop a model for the propellant transition for one product and subsequently re-use the model for other products with minor adjustments. Here, one can see that an MMF could have the advantage of avoiding repeated documentation and review. Furthermore, the modular nature of the workflow (i.e., CFD, PBBM, etc.) would also allow for having separate MMFs describing each module separately. This could simplify updating at later times and also help to guide the sponsor and reviewer to identifying which elements of a model that are likely adequate as presented (and therefore in no need for repeated review), and which elements that would require more rigorous review.

### Modeling Methodologies Integrating Diverse Data Sets to Support the Development and Approval of Dermatological Products

**Abdullah Hamadeh, PhD**

**University of Waterloo**

As described in a presentation by Dr. Abdullah Hamadeh (University of Waterloo), an MMF for a PBPK model may be viewed as a validated compendium of the absorption, distribution, metabolism, and elimination (ADME) mechanisms governing drug PK [37]. Its value lies in its acceptance as a reference mathematical model structure with associated parameter values, all backed by experimental data and documentation. In the specific context of developing an MMF of dermal drug absorption, a mechanistic understanding of multiple factors that change with each application context is required [38, 39].

Construction of a useful PBPK for drug products applied to the skin (i.e., a dermal PBPK model) requires consideration of many factors. Alongside the physical and chemical characteristics of the permeating compound, its transport across the skin barrier depends on skin physiology, skin anatomy, excipient effects, the applied dose, and ambient conditions. To account for these factors, dermal models have evolved from empirical relationships linking a drug’s skin permeability with its physical/chemical characteristics [40–42] to mechanistic representations of skin diffusion captured via partial differential equations [43–46]. Altogether, the robustness of dermal PBPK model predictions requires that: 1) the model structure accurately represents the physical processes underpinning skin permeation, 2) model parameters are known with a degree of precision that allows for informative predictions, and 3) the variability in parameters that are specific to populations, individuals and anatomical sites is quantified [38]. Fortunately, a wealth of experimental methods is available to derive this information.

Dr. Hamadeh described an example of a dermal PBPK model supported by *in vitro* and *in vivo* studies. In this example, large-scale *in vitro* studies have been conducted to measure skin penetration and accumulation [47], the partitioning and diffusivity of compounds in the different skin layers [48], and the rate of permeant metabolism in skin [49] for numerous compounds. The compounds used in these studies cover a broad range of physical and chemical properties, which enabled the use of statistical learning tools to infer relationships between the descriptors of chemicals and their rates of transport across the various skin strata [50]. In addition, these experiments have been conducted on multiple skin sections sourced from various individuals, allowing for the elucidation of variability both between and within individuals using the same learning methods. *In vivo* studies may be employed to measure systemic exposure to dermally applied chemicals via plasma measurements, as well as local exposure via stratum corneum tape stripping experiments for further model validation [51, 52]. When combined with *in vitro* skin permeation tests of the same formulations, these experiments can be used to develop an understanding of formulation effects on skin absorption under real-world conditions [39].

Dr. Hamadeh noted that integrating this accumulated knowledge into a reference MMF requires a robust validation framework combining several key components including: 1) databases of measurements from experimental studies, 2) databases detailing the exact context of these studies (e.g., compound, vehicle, exposure conditions, skin conditions) to ensure accurate model configuration, 3) databases of measured or estimated model parameters, including their uncertainties, variabilities and correlations, 4) a well-documented mechanistic model structure, 5) extensive simulations of the model to demonstrate its ability to accurately capture observed dermal absorption measurements and their variabilities. By incorporating these elements, the framework can ensure that the MMF is a well-grounded and reliable resource for the simulation of dermal administration scenarios of practical relevance.

### Development and Verification of Mechanistic Dermal Absorption Models for Submission in a Model Master File

**James F. Clarke, PhD**

**Certara**

**Fig. 4:**
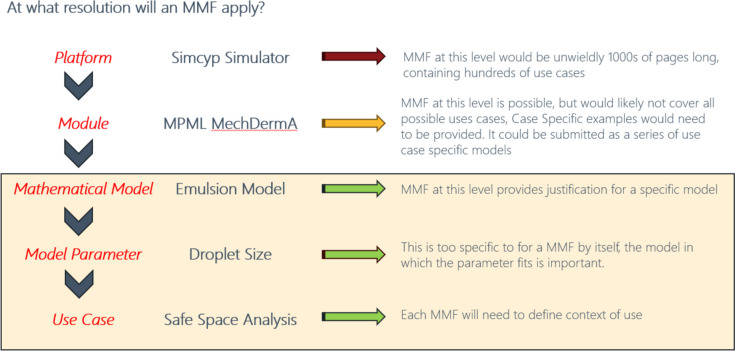
Hierarchy that describes potential utility of an MMF for the MPML MechDermA module within the Simcyp Simulator, that considers an MMF at the level of the overall software platform down to the level of a use case. In this case, “safe space analysis” refers to the impact of differences in droplet size as incorporated into an emulsion model on the predicted metrics of interest that may relate to local tissue PK or systemic PK.

Dr. James F. Clarke (Certara) delivered a presentation focused on considerations for an MMF in the context of dermal absorption and challenges from the perspective of a software vendor [53]. He began by outlining a hierarchy for MMF submissions, considering the resolution at which an MMF may be applicable. Figure 4 shows the different levels discussed in this hierarchy, starting at the level of an entire modeling framework (software platform), and focusing down into a specific use case. Dr. Clarke proposed that each use case will need to be set within a COU and therefore will need to include, or reference, information on the model in which it is to be used. However, due to the size and complexity of some models, using the multi-phase multi-layer (MPML) mechanistic dermal absorption (MechDermA) module (Certara, Inc., Radnor, PA, USA) and a software platform such as the Simcyp Simulator (Certara, Inc., Radnor, PA, USA) as examples, it may not be appropriate to have an MMF at these levels. Instead, the ideal scenario would likely include several MMFs for each module or mathematical model, each focusing on a particular use case.

Using the example of an emulsion formulation safe space analysis, the potential information that may be included in an MMF was discussed. Regarding the description of the model and its features, it was suggested that published work may only need to be cited, but non-published or proprietary works would need to be described in more detail within the MMF for proper assessment. It was, however, unclear at the time of the presentation how much detail may yet be needed in the MMF.

Some potential use cases for an MMF were provided, such as an MMF demonstrating the MPML MechDermA’s model ability to extrapolate predictions to a pediatric population or to predict local concentrations. Dr. Clarke then elaborated on the example of the diclofenac gel submission and discussed how the submission may have been done differently within an MMF framework. Dr. Clarke proposed that the verification report (discussed in [10]), with some small modifications, could be submitted as an MMF if the pathway was available. Finally, Dr. Clarke discussed challenges from the perspective of a software vendor. As the Simcyp Simulator has a one year release cycle, this may present issues with versioning when referencing an MMF. It was proposed that there should be a simple process available to bridge between software version for an MMF without having to completely resubmit the MMF.

## Panel Discussion

Drs. Sujatha Sonti (GSK) and Khondoker Alam (FDA) moderated a panel discussion to close the session, which included the speakers previously introduced in this article as well as Mr. Jay Mowli (Capstone Development Services Co, LLC), Dr. Jessica Spires (Simulations Plus, Inc.), and Dr. Ming-Liang Tan (FDA). The conversation was kicked off by Dr. Walenga, who responded to a question on techniques for validating predictions of local tissue concentrations for topically delivered drug products such as OIDPs, ophthalmic drug products, and drug products applied to the skin. Dr. Walenga replied that for OIDPs, if the goal of the model is to predict regional deposition, then *in vivo* nuclear imaging methods such as gamma scintigraphy, single photon emission computed tomography (SPECT)/CT, and positron emission tomography (PET)/CT may be considered. However, if the purpose of the model is to predict lung tissue concentration, there are a limited number of bronchoalveolar lavage studies that have quantified lung tissue concentration that are rare and invasive. Thus, a bracketing approach may be more appropriate, where model predictions of regional deposition and systemic PK are both validated. For ophthalmic products, Dr. Tan commented that validation is challenging because not only are local tissue concentration values typically unavailable, but systemic PK is often difficult to quantify. For ophthalmic drug products, aqueous humor PK data for model validation may be collected in cataract patients undergoing surgery. Additionally, for products that are indicated for lowering intraocular pressure (IOP), differences in IOP can be quantified and incorporated into a PD model that may then be coupled to a PK model. For drug products applied to the skin, Dr. Clarke said that there are some *in vivo* microdialysis data available for model validation, and IVPT data may be useful as well, but it is still unclear how well IVPT data correlate with *in vivo* tissue concentration in the skin. It may be a viable approach to leverage quantitative structure–activity relationship (QSAR) modeling along with drug physicochemical properties to parameterize the PBPK model that is optimized against observed *in vivo* microdialysis and systemic PK data for one active ingredient. If that model validation is favorable, an MMF may then be applied for other active ingredients such that validation with *in vivo* tissue concentration data may not always be needed. Dr. Hamadeh added that one method for validating dermal PBPK models in a comprehensive manner would be a combination of *in vivo* tape stripping data, *in vitro* tissue permeation data, and systemic PK data. Dr. Fridén expressed that for all locally acting routes of administration that there will always be challenges associated with making robust experimental determination of local exposure. However, regardless of those challenges, using any relevant *in vivo* data to validate the model should increase confidence.

Mr. Mowli proposed that because an MMF application depends a lot on the COU and any firm that is using the model will likely generate their own proprietary validation data set for the specific compound of interest, it may be more useful, from a model reusability perspective, to apply the MMF concept for validation of a modeling framework (software platform). Dr. Musuamba noted that from a regulatory point of view, the confidentiality of the data should not be an issue since the regulatory agency would be working under a confidentiality agreement.

The next question posed to the panel by Dr. Sonti was whether a PBPK model may be represented by two MMFs if it includes model predictions for both *in vitro* and *in vivo* outcomes. With respect to employing *in vitro* and *in vivo* data towards development and validation of mechanistic models for OIDPs and skin products, Drs. Clarke and De Backer, respectively, agreed that both types of data would be beneficial especially in the case of establishing IVIVEs. An MMF may encompass IVPT data for a dermal PBPK model or APSD and patient breathing profile data for a mechanistic model of an OIDP, serving in this way as a useful guideline for what model elements can be selected from various sources. Along these lines, Dr. Hamadeh said that the model could be split up into different components, such as the physiological and formulation aspects. Dr. Spires offered that the decision to use one or multiple MMFs may depend on whether the model is constructed with the help of a CRO or if it is constructed completely in-house. However, an in-house model may also be split up into multiple MMFs that capture specific aspects of the overall approach. Dr. Walenga noted that in the case where an IVPT model for a skin product is not expected to require significant updates or has received regulatory acceptance, can therefore be submitted under an MMF while the *in vivo* model for the same product may need to be submitted under an actual submission due to model updates, depending on the availability of new data. To provide further context, Dr. Alam pointed out that several MMFs or subsections of MMFs may be referenced by the same application to form a cohesive modeling approach under the consideration of the COU for the referenced MMFs.

Drs. Andrew Hooker (Uppsala University), Musuamba, and De Backer, discussed the need for an MMF holder to clearly state the COU for a submitted MMF and the relevancy of the COU of the MMF when assessed within the scope of a regulatory application that references the said MMF. This is similar to the approach taken by EMA in regard to the Qualification Opinion and Qualification Advice program and consistent to the application of M&S approaches supporting regulatory submissions.

Drs. De Backer, Sonti, Tan, and Walenga expressed the need for a meeting pathway, potentially though the newly established Quantitative Medicine Center for Excellence [54], between industry/CROs and FDA that would foster discussions on the development of an MMF prior to submission. The panelists agreed that establishing a forum for communication between FDA and interested parties will facilitate the implementation of the MMF in a more efficient manner. Dr. Sonti also suggested that it may be valuable to communicate success stories to encourage future MMF submissions.

Dr. Essam Kerwash (Medicines and Healthcare products Regulatory Agency [MHRA]) asked about whether OIDP models are specific to a certain active ingredient or if they can be applied for many active ingredients under the consideration that certain aspects of these model can be reported under an MMF referenced across multiple regulatory applications. Dr. Fridén replied that OIDP models may be modified for certain characteristics of the active ingredients to perform better and Dr. De Backer added that the device, inhalation profile, APSD, and disease lung model that typically drive deposition predictions, but that the key challenge is quantifying variability, especially when applied to a virtual clinical trial.

## Small Group Discussion

During the associated small group discussion, the workshop attendees agreed that the MMF initiative holds the potential to decrease the effort and resources that are necessary to develop an M&S approach supporting a regulatory submission since the same MMF or part of an MMF can be reused across multiple applications. Locally acting drug products, considered complex in the generic space, may benefit from an M&S approach that includes an MMF that aims to address challenges associated with their development and regulatory approval. Several potential applications were discussed, including an adequately validated model that describes local drug exposure and its relationship with systemic drug exposure. For drug-device combination products, it was suggested that measuring drug concentration at start and end points within the delivery device may strengthen the validation process. With respect to OIDPs, participants proposed that integration of CFD and PBPK models into a single MMF may be a promising M&S approach that could promote the development of these products by conducting risk assessments to manage and mitigate potential issues that may occur during product development and may require formulation and/or device changes. The workshop attendees agreed that developing distinct MMFs tailored to the unique requirements of different inhalation product types, such as DPIs and MDIs, may be extremely beneficial across multiple products in the pipeline. For all locally acting drug products where the site of action may not be accessible, the application of PBPK/PD models that allow for predictions at the site of pharmacological action were proposed as MMFs that could help overcoming challenges with assessing *in vivo* performance for these products. Modeling framework (software platform validation) was also considered as a potential MMF for locally acting drug products. Although many promising applications were proposed, the workshop attendees noted that science is still developing for these drug products, and that the relevant in silico models may yet not be mature enough.

## Conclusion

The MMF concept has previously been introduced as a means for protecting proprietary M&S information and intellectual property within the context of drug applications to FDA. The workshop session described in this work was organized to explore the potential use of MMFs for mechanistic models of locally acting drug products that may support drug development and approval. The six presentations in this workshop session described methods for building effective mechanistic models of locally acting drug products, discussed what information may need to be included in an MMF for such models, considered versioning for MMFs when changes are needed, and introduced potential applications for MMFs of mechanistic models for OIDPs, drug products applied to the skin, and ophthalmic drug products. Additionally, a presentation from Dr. Musuamba described the Qualification of Novel Methodologies program that EMA uses to provide feedback to requestors regarding the use of novel methodologies for supporting drug product development, which provided a useful point of comparison and contrast with the MMF concept. The panel discussion covered topics that included *in vivo* validation of local tissue concentration predictions, the use of multiple MMFs to describe components of a holistic modeling approach, and potential efficiency enhancements and challenges associated with MMFs. The subsequent small group discussion session yielded agreement on the potential reduction in resources that may be associated with the MMF concept. Also, the workshop attendees agreed that a promising application for MMFs of locally acting drug products may be a mechanistic model that can describe relationships between local tissue concentration and systemic concentration values. By exploring potential benefits and challenges, the workshop session served to educate and stimulate the pharmaceutical industry, FDA, and academia on the use of the MMF concept to support drug development and approval for locally acting drug products.

## Data Availability

Data sharing not applicable to this article as no datasets were generated or analysed during the current study.

## Declarations

### Disclaimer

The article reflects the views of the authors and should not be construed to represent their organizations’ views or policies.
